# Glucogenic Precursor Release from Dietary Supply Is a Potential Amplifier of Monosodium-Glutamate Ovary Stimuli in Sheep with Low Involving Key Gene Mediators of the Glutamate Pathway

**DOI:** 10.3390/ani15162345

**Published:** 2025-08-11

**Authors:** Yohana Huicho Miguel, Juliana Paula Martins Alves, Ana Flávia Bezerra da Silva, Alfredo José Herrera Conde, Camila Muniz Cavalcanti, Louhanna Pinheiro Rodrigues Teixeira, Jhennyfe Nobre de Sena, Fernando Felipe da Silva Pereira, César Carneiro Linhares Fernandes, Dárcio Ítalo Alves Teixeira, Davide Rondina

**Affiliations:** 1 School of Veterinary Medicine, State University of Ceará, Fortaleza 60714-903, CE, Brazil; yohana.huicho@aluno.uece.br (Y.H.M.);; 2 Experimental Biology Center, University of Fortaleza, Fortaleza 60811-905, CE, Brazil; 3 Health Sciences Center, University of Fortaleza, Fortaleza 60811-905, CE, Brazil

**Keywords:** sheep, ovary, follicles, monosodium glutamate, glycerin, luteal function

## Abstract

This study aimed to evaluate the effect of combined supplementation of MSG with glycerin, a glucogenic precursor, on ovarian function in sheep. Twenty-four ewes had estrus and follicular waves synchronized using three prostaglandin injections at 7-day intervals. The ewes were grouped: baseline TMR diet (Control; *n* = 8); glutamate diet (MSG; *n* = 8), receiving MSG (1 g/kg of body weight/day) for 16 days; and MSG plus glycerin (MSGLY; *n* = 8), which received MSG plus 150 mL of glycerin during the eight days prior to ovulation induction. MSG showed lower dry matter intake, while the MSGLY group showed increased heart and respiratory rates and skin temperature. Rectal temperature was higher in MSG and MSGLY. MSGLY also showed reduced triglyceride and urea levels. MSG and MSGLY exhibited decreased cholesterol and creatinine. MSGLY exhibited a higher number of large follicles and greater intraovarian blood perfusion after ovulation induction and larger corpus luteum perfusion. Ovulation rate increased by 64% in the supplemented groups vs. control. MSG supplementation led to greater SCL1A1, GRIA1, and GLUD1 genes expression. Thus, the combined supplementation of MSG and glycerin effectively enhances ovarian function in sheep, representing a viable nutritional strategy to improve reproductive outcomes.

## Simple Summary

Monosodium glutamate (MSG) has been shown to be a promising nutritional supplement for goats and sheep to support ovarian and reproductive function. MSG, associated with the neuroexcitatory effect of the amino acid and its low ruminal degradability, allows it to be administered directly to ruminant feed, which opens opportunities for its use in the field for producers. Efficacy of glutamate depends on multiple factors, the main one being energy availability. However, the impact of these potential interactions on ovarian response in ruminants remains unclear. The present study showed in sheep that supplementation combined with MSG and glycerin, the latter product acting as a rapid glucose releaser, stimulates follicular growth and intraovarian blood flow, boosting the ovulation rate.

## 1. Introduction

Among amino acids, glutamate supplementation has emerged as an effective and promising nutritional strategy to improve reproductive efficiency in ruminants [1], particularly due to its neuroexcitatory and stimulatory effects on ovarian function [2]. In goats, intravenous administration of glutamate stimulates follicular development and ovarian response in animals with low body condition [3] or anovulation [4]. Its partial resistance to ruminal degradation [5] has recently enabled the use of glutamate in the form of monosodium salt (MSG) directly in feed, expanding its practicality and feasibility as a field-level supplement for producers. In goats, MSG has been shown to enhance follicular growth and intraovarian blood perfusion [6]. In sheep, oral administration has resulted in a higher proportion of animals exhibiting estrus and pregnancy [7].

Although studies in rodents supplemented with oral glutamate for five weeks have shown that it improves the locomotor performance and cognitive function of the animals, which can be attributed to an improvement in the antioxidant status and in the cholinergic, monoaminergic and glutamatergic neurotransmission in the brain and hippocampus [8], its application in ruminants remains underexplored and faces several challenges, particularly due to the complex interactions among diet, ruminal microbiota, and energy metabolism [1]. The action of glutamate on ovarian function is multifactorial, modulating both molecular and metabolic signaling pathways. Glutamate interacts with ionotropic (NMDA, AMPA, and kainate) and metabotropic (mGluRs) receptors, regulating the secretion of gonadotropins such as FSH and LH, which are essential for follicular growth and ovulation [4]. High glutamate levels are also associated with increased prostaglandin E2 transmission, which amplifies GnRH secretion [9], reinforcing glutamate’s role in reproductive processes. Glutamate is also a precursor of alpha-ketoglutarate, a key intermediate in the tricarboxylic acid cycle [1], essential for supplying energy to ovarian follicles. Furthermore, it contributes to nitric oxide production, which is crucial for ovarian blood flow and maintaining follicular quality [10]. Recent studies show that glutamate regulates oxidative stress [8] and affects epigenetic mechanisms [11], both of which are fundamental for follicular development. Energy availability plays a central role in this scenario.

Under conditions of energy restriction, the metabolism of glutamate and other amino acids may be compromised, leading to insufficient availability for ovarian function. Glutamate is crucial for protein synthesis and energy metabolism, which are essential for reproductive health [1]. Studies indicate that glutamate supplementation can stimulate follicular development and increase intraovarian blood flow, which is essential for the health of developing follicles. In goats with low body condition scores, glutamate improved follicle number and size, indicating increased ovarian response [3]. Studies suggest that energy supplementation enhances glutamate’s action, promoting more efficient follicular activation and reducing atresia [4]. However, dietary manipulation in ruminants is challenging due to nutrient degradation and interaction with the ruminal microbiota, which can affect compound bioavailability [1]. The combination of bioavailable supplements capable of overcoming these limitations and effectively optimizing ovarian function is a key strategy to enhance glutamate’s effects. Among energy supplements, glycerin undoubtedly stands out due to its availability [12], rapid ruminal degradation and glucose release [13], and proven positive effects on ovarian function, even with short administration intervals [14,15].

Despite the known reproductive actions of glutamate and glycerin, little is understood about the interactions between these compounds and their regulation of key genes involved in energy and reproductive metabolism, or how glutamate supplementation and energy availability modulate these processes. Investigating the expression of these genes is essential to understand how the diet can optimize ovarian/reproductive function. Circulating glutamate promotes the expression of N-methyl-D-aspartate (NMDA; GRIA1) and α-amino-3-hydroxy-5-methyl-4-isoxazolepropionic acid (AMPA) receptors [16] in GnRH [17] and glutamatergic KNDy neurons [18], enabling pulsatile GnRH release. Exogenous glutamate also stimulates insulin production, as NMDA, AMPA, and kainate receptors are present in pancreatic α and β cells, allowing glutamate to influence pancreatic function [4]. The insulin produced subsequently stimulates GnRH production through receptors located in the hypothalamic–pituitary–gonadal axis [19]. Moreover, the GLUT4 and GRIA1 genes are respectively responsible for glucose transport into cells [20] and modulation of the follicular response to glutamate [21], which is converted into α-ketoglutarate by glutamate dehydrogenase 1 (GLUD1), thereby supplying energy directly to ovarian follicles [22].

In this context, the central hypothesis of this study is that glutamate’s stimulatory effect on ovarian function in sheep can be optimized by ensuring adequate dietary energy availability. Therefore, this study aimed to investigate the effects of combined dietary supplementation with monosodium glutamate and glycerin, the latter serving as a glucogenic precursor, on ovarian follicular development, intraovarian blood perfusion, and luteal function in sheep. Additionally, the study examined the expression of genes related to glutamate and energy metabolism, as well as physiological responses and the plasma biochemical profile during the supplementation period.

## 2. Materials and Methods

### 2.1. Ethic Statements and Location Facility

All procedures involving animals were approved by the Animal Use Ethics Committee of the Ceará State University (NUP 31032.005729/2023-23). The trial was conducted at the facilities of the Ruminant Nutrition and Husbandry Laboratory, part of the Veterinary School of Ceará State University, Brazil. The site is located in the equatorial zone (4°2′23″ S and 38°38′1″ W), ensuring full compliance with ethical and scientific standards.

### 2.2. Animals, Feeding and Housing Management, and Pre-Experimental Conditions

Twenty-four adult, non-lactating, non-pregnant Santa Inês sheep were selected for the trial. At the beginning of the experiment, the ewes were weighed, assigned the body condition score, and adipose and muscle masses were measured by ultrasonography to assess subcutaneous fat thickness over the loin, depth of the loin muscle, and perirenal fat, according to Wang et al. [23]. The sheep BMIs were calculated using the following formula: BMI = ((Body weight [kg]/Height at withers [m]/Body length [m])/10). The overall means (±SD) for age, body weight, height at withers, body length, and body condition score were 34.9 ± 9.6 months, 42.1 ± 6.7 kg, 68.1 ± 4.0 cm, 69.2 ± 4.3 cm, and 2.8 ± 0.1, respectively. The ewes were grouped based on their body mass index (BMI) and kept in collective covered pens with concrete floors, where they received water and mineral salt ad libitum. Each box was allocated at least 2 or 3 sheep, each constituting a subgroup.

All females were fed with the same diet consisted of a total mixed ration (TMR) composed of fresh, chopped elephant grass (*Pennisetum purpureum* spp.) and concentrate feed with a vitamin/mineral supplement, provided in quantities based on the nutritional requirements of breeding adult sheep [24]. Feed was offered in two daily meals at 08:00 h and 15:00 h, and intake was monitored daily during the experimental period. Refusals were maintained at 10%, and the particle size of the TMR was assessed using a Penn State particle separator, following the methodology described by Heinrichs and Kononoff [25]. Before the trial began, the animals underwent a 30-day adaptation period in the housing facility, during which they received endo- and ectoparasite treatments and were vaccinated against clostridiosis. Throughout the pre-experimental period, cyclicity and ovarian function were monitored by ultrasound examinations and sexual receptivity to a fertile, mature ram, following the protocol described by Fernandes et al. [26].

### 2.3. Experimental Design

**Figure 1:**
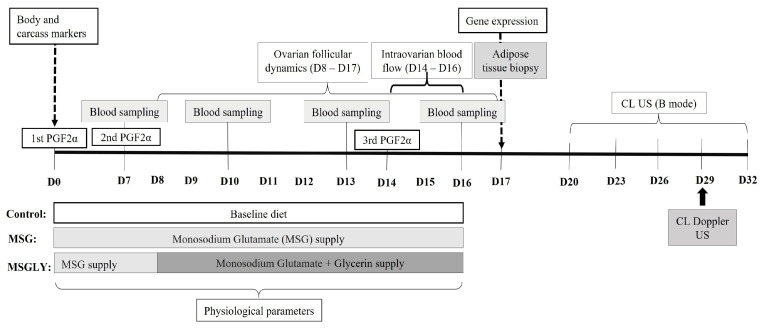
Timeline of experimental design, describing, sampling and measures periods, the intervals of nutritional treatments, and hormonal protocol applied for estrus and follicular wave synchronization.

All ewes were subjected to a hormonal protocol for the induction and synchronization of estrus and follicular waves according to the ‘first wave’ methodology proposed by Viñoles et al. [27], in order to evaluate the nutritional effects on ovarian follicular dynamics. Three injections of 0.263 mg of a prostaglandin analogue (Cloprostenol sodium, Sincrocio^®^, Ourofino, São Paulo, Brazil) were administered at 7-day intervals (Figure 1). The day of the first prostaglandin (PGF2α) injection was considered day 0 (D0, Figure 1).

On D0, the ewes subgroups were randomly assigned into three experimental diet-groups, homogeneous for BMI and fat and muscle mass (Table 3): Control (*n* = 8), which received only the baseline diet; Glutamate group (MSG; *n* = 8), which received the baseline diet plus monosodium glutamate (1 g/kg of body weight/day) from D0 to D16; and Glutamate plus Glycerin group (MSGLY; *n* = 8), which received the MSG diet from D0 to D16, with the addition of 150 mL of glycerin daily from D8 to D16. Monosodium glutamate (Della Terra^®^, São Paulo, Brazil, 99% purity) was incorporated directly into the concentrate feed before preparation of the TMR. Glycerin (UPS, Lucas Pires Chemical Products LTDA, Mossoró, Brazil, 99% purity) was administered as a water-based solution (9:1 glycerin:water), mixed directly into the TMR. To facilitate mixing of the TMR ingredients and ensure similar moisture content across diets, a water solution was added to the TMR of the Control and MSG groups, which was based on the energy density of glycerol (0.38 Mcal of ME/mol) [28].

### 2.4. Assessment of Ovarian Function Outcomes

#### 2.4.1. Follicular Dynamics

Ovarian follicular dynamics were assessed daily using B-mode ultrasonography (DP-2200Vet, Mindray Bio-Medical Electronics Co., LTD., Shenzhen, China), with a 5 MHz transrectal linear probe. Evaluations began on day 8 (D8) of the hormonal protocol and continued until day 17 (D17). On days 14, 15, and 16, ultrasound examinations were performed every 12 h (morning and afternoon) to increase accuracy in monitoring follicular dynamics. Each ovary was video recorded for later analysis and follicle measurement using ImageJ^®^ software (V.1.54g, National Institutes of Health, Millersville, PA, USA), previously calibrated. Examinations were performed in the morning before feeding, with the animals standing. Feces were removed from the rectum, and the transducer was inserted using lubricating gel as a contact agent. The probe was moved laterally to visualize both ovaries, allowing the observation and counting of follicles. An ovarian follicular wave was defined as the emergence of a group of small follicles (<3 mm) that developed into one or more large follicles (≥3 mm). The day on which the largest follicle of the wave reached 3 mm in diameter was defined as the day of wave emergence. The growth phase was the period during which a large follicle grew from 3 mm to its maximum diameter. The regression phase was defined as the period from the maximum diameter back to 3 mm.

#### 2.4.2. Intraovarian Blood Perfusion

Intraovarian blood flow was assessed on days 14, 15, and 16 (Figure 1) using ultrasound videos of both the right and left ovaries captured with color Doppler mode (Model M6Vet, Mindray Animal Care Bio-Medical Electronics Co. Ltd., Shenzhen, China). The 5 MHz transrectal linear probe was set with a pulse repetition frequency of 1.0 kHz, a depth of 6.5 cm, and a color gain of 60%, maintaining consistent settings throughout the evaluations. Videos were analyzed quantitatively using ImageJ^®^ software (V.1.54g, National Institutes of Health, Millersville, PA, USA), focusing on the colored pixels that represented blood flow. Briefly, the cross-sectional area of the ovary showing the most intense Doppler signal was selected, and two areas were manually outlined: the total ovarian area (TA), representing the ovarian surface, and the Doppler area (DA), corresponding to the visible blood flow. The percentage of the area with blood perfusion was calculated as (DA/TA × 100%) for each ovary, following the method described by Oliveira et al. [29].

#### 2.4.3. Corpus Luteum Growth, Luteal Blood Perfusion Area, and Ovulatory Rate

Corpus luteum (CL) development and regression were monitored every three days from days 20 to 32 (Figure 1) using B-mode ultrasonography (DP-2200Vet, Mindray Bio-Medical Electronics Co., Ltd., Shenzhen, China). Videos were captured and analyzed using ImageJ^®^ software, which enabled measurement of CL diameter. Ovulation rate was assessed nine days after the third PGF2α administration, as described by Viñoles et al. [27]. Ovulation was confirmed by the collapse of ovulatory follicles and the presence of CL.

On day 29, corresponding to the 15th day after ovulation induction (Figure 1), CL vascularization was assessed. A color Doppler ultrasound device (Model M6Vet, Mindray Animal Care Bio-Medical Electronics Co., Shenzhen, China) equipped with a 5.0 MHz transrectal linear array transducer was used. The CL was initially identified in B-mode, and additional color Doppler videos were recorded to assess vascularization. The image showing the largest cross-section of the CL was selected. Manual delimitation of the CL and colored areas was performed in ImageJ^®^ software using the freehand selections tool. The total area of the CL and the Doppler area were calculated to determine the percentage of vascularization (% Doppler area), according to Balaro et al. [30].

### 2.5. Physiological Effort During the Period of Dietary Supplementation

The animals’ physiological responses were assessed twice daily, at 07:00 and 14:00, from day 0 to day 16 (Figure 1). Rectal temperature (RT) was measured using a digital clinical thermometer (G Tech^®^, Hangzhou Sejoy Electronics, Hangzhou, China). Skin surface temperature (ST) was assessed at a previously sheared area on the rump using an infrared thermometer (AK32^®^, AKSO, São Leopoldo, Brazil). Heart rate (HR) was measured with the animal standing, using a stethoscope (3M Littmann^®^, Master Classic II™, St. Paul, MN, USA) placed on the left side of the thorax near the heart. Pulses were counted for 15 s and multiplied by four to calculate beats per minute. Respiratory rate (RR) was measured by auscultation of lung sounds for 15 s, and the resulting value was also multiplied by four.

### 2.6. Blood Sampling and Metabolite Assay

Blood samples were collected from the jugular vein every three days starting from the second PGF2α application (Figure 1), using heparinized tubes (FIRSTLAB^®^, Disera Tıbbi Malzeme, İzmir, Turkey). Samples were centrifuged (907× *g*, 10 min), and plasma was stored at −20 °C for the analysis of total protein, glucose, cholesterol, triglycerides, creatinine, and urea. Analyses were conducted using an automated biochemistry analyzer (Mindray^®^ BS 120, Mindray Biomedical Electronics Co., Shenzhen, China) and commercial kits (Bioclin, Quibasa, Minas Gerais, Brazil). Kit sensitivities and intra- and inter-assay coefficients of variation (CV) were as follows: (1) Total protein: sensitivity = 0.043 g/dL; intra- and inter-assay CV = 0.46% and 2.24%; (2) Glucose: sensitivity = 1.31 mg/dL; intra- and inter-assay CV = 2.59% and 0.78%; (3) Cholesterol: sensitivity = 1.472 mg/dL; intra- and inter-assay CV = 1.35% and 1.85%; (4) Triglycerides: sensitivity = 2.58 mg/dL; intra- and inter-assay CV = 0.59% and 0.54%; (5) Urea: sensitivity = 1.514 mg/dL; intra- and inter-assay CV = 2.96% and 1.17%; and (6) Creatinine: sensitivity = 0.034 mg/dL; intra- and inter-assay CV = 0.89% and 1.06%.

### 2.7. Adipose Tissue Sample Collection

Subcutaneous adipose tissue samples were collected by biopsy at the base of the ewes’ tails on day 17, three days after ovulation induction. The procedure was performed under sedation with xylazine (0.2 mg/kg, i.m., 2% Anasedan^®^, Ceva, São Paulo, Brazil) and local anesthesia with lidocaine (3 mL, Lidovet^®^, BRAVET, Rio de Janeiro, Brazil). After asepsis and hair removal, a 3 cm incision was made to collect approximately 2 g of adipose tissue. The samples were rinsed with Milli-Q water, weighed, stored in cryovials submerged in liquid nitrogen, and preserved at −80 °C for subsequent RNA extraction.

### 2.8. RNA Isolation and Reverse Transcription Real-Time Quantitative Polymerase Chain Reaction (RT-qPCR) of Glutamate and Energy Genetic Markers

Total RNA was extracted using Trizol^®^ reagent (Invitrogen, Carlsbad, CA, USA), and RNA concentration was determined with a NanoDrop^®^ 2000 spectrophotometer (Thermo Fisher Scientific, Waltham, MA, USA). For cDNA synthesis, 1 µg of total RNA was used with the High-Capacity Reverse Transcription Kit (Thermo Fisher Scientific^®^, Vilnius, Lithuania). The abundance of mRNA for candidate genes (Table 1) related to amino acid transporters (SCL1A1, SCL1A3), glutamate metabolism (GRIA1, GLUD1), and glucose and energy regulation (GLUT4, LEPTIN) was assessed by qPCR using the StepOnePlus™ Real-Time PCR System (Applied Biosystems^®^, Foster City, CA, USA) with Power SYBR^®^ Green PCR Master Mix (Invitrogen^®^, Warrington, UK). The RPS18 gene was used as an endogenous control for normalization. Primers were designed using the Primer-BLAST tool from NCBI GenBank, specific for *Ovis aries* (Table 1). Amplification specificity was confirmed by dissociation curve analysis. Table 2 details the thermal cycling conditions used in the RT-qPCR reactions. The ΔΔCT method [31] was used to convert cycle threshold (Ct) values into normalized relative mRNA expression levels.

### 2.9. Data Statistics and Analysis

All statistical analyses were performed using Statistica Software version 13.4.0.14 (2018; TIBCO Software, Inc., Palo Alto, CA, USA). Normality was assessed using the Shapiro–Wilk test, and non-normally distributed data were log-transformed. Data were analyzed using ANOVA with GLM procedures in a factorial model. For physiological parameters, the factors included group (Control, MSG, MSGLY), time (week 1, week 2), period of reading (morning, afternoon), and interactions between group and time or period of reading. For dry matter intake, metabolic parameters, follicular dynamics, and intraovarian perfusion area, the factors included group, sampling interval (time), and interaction between group and time. Finally, for body and carcass traits, luteal blood perfusion, and RNA expression, the factor was group only. Differences between means were evaluated using the Newman–Keuls post hoc test when ANOVA indicated a significant effect (*p* < 0.05).

## 3. Results

### 3.1. Feed Intake

There was a significant effect on both feed intake parameters (Table 3), with a notable decrease observed in the second week of the trial, following the second PGF2α administration in the MSG and MSGLY groups (time effect, *p* < 0.001). Regarding the group effect (Table 3), animals supplemented with glutamate showed lower dry matter intake (*p* < 0.05) compared to the other treatments. Despite this, intake levels throughout the trial remained above the expected values (>2.0% BW; Table 3). Based on intake data, the glycerol dose used in the MSGLY group represented a 43% increase in the energy requirement.

**Table 3:** Body and carcass markers, feed intake, physiological effort, and metabolic effort in ewes fed with baseline diet (Control) or supplemented with glutamate monosodium (MSG), or MSG plus glycerin (MSGLY).

Parameters	Group				*p*-Value				
	Control	MSG	MSGLY	SEM	Group	Time	DR	G vs. T	G vs. DR
Body and carcass markers *									
BMI	8.6	9.0	8.7	0.205	0.186	-	-	-	-
SLFT, mm	4.1	5.1	4.2	0.366	0.350	-	-	-	-
KFT, mm	2.2	2.3	2.5	0.103	0.682	-	-	-	-
LD, mm	17.4	19.4	17.9	0.884	0.122	-	-	-	-
*Feed intake*									
DMI, g/MW	68.3 ^a^	61.2 ^b^	67.2 ^a^	0.973	0.001	<0.001	-	0.136	-
DMI, % BW	2.7 ^a^	2.4 ^b^	2.7 ^a^	0.042	<0.001	<0.001	-	0.192	-
*Physiological effort*									
Rectal temperature, °C	38.1 ^a^	38.3 ^b^	38.3 ^b^	0.020	<0.001	<0.001	<0.001	<0.001	0.806
Surface temperature, °C	34.0 ^a^	34.0 ^a^	34.6 ^b^	0.050	<0.001	<0.001	<0.001	0.451	0.007
Heart rate, beats/min	70.3 ^a^	70.6 ^a^	72.5 ^b^	0.426	0.024	0.039	<0.001	0.002	0.016
Respiratory rate, breaths/min	32.1 ^a^	37.5 ^b^	40.1 ^c^	0.667	<0.001	<0.001	<0.001	<0.001	0.103
*Metabolic effort*									
Glucose, mg/dL	62.3	60.9	60.6	0.513	0.363	0.590	-	0.288	-
Total Protein, mg/dL	6.4	6.1	6.2	0.058	0.083	0.854	-	0.052	-

* Performed at the beginning of the experiment; BMI, body mass index; SLFT, subcutaneous loin fat thickness; KFT, kidney fat thickness; LD, loin depth; DMI, dry matter intake; MW, metabolic weight. The *p*-value for the ANOVA effects for group, effect for interval of sample used (effect time), effect for daily reading measures (DR: morning, afternoon), and interaction group vs. time, group vs. daily reading are shown in the table; ^a,b,c^, *p* < 0.05 differences between groups.

### 3.2. Physiological and Metabolic Efforts

The MSG and MSGLY groups exhibited higher values (*p* < 0.05) than the control group for rectal temperature and respiratory rate (Table 3). Animals supplemented with glycerin recorded higher values for all physiological parameters compared to the other treatments (*p* < 0.05).

All measurements (Table 3) showed a significant increase in the afternoon (period-of-reading effect, *p* < 0.001) and after the second PGF2α administration (time effect, *p* < 0.05). A significant interaction between group and period of reading was observed for heart rate (*p* = 0.016) and surface temperature (*p* = 0.007), driven by the significantly higher MSGLY values recorded in the afternoon. Except for surface temperature, all other parameters showed a significant interaction (*p* < 0.01) between group and supplementation period. In this case, morning readings were similar between groups, whereas in the afternoon, heart rate was higher in MSGLY than in the other treatments (75.0 ± 1.1 beats/min vs. 70.3 ± 1.0 beats/min; *p* < 0.001). For rectal temperature, both morning and afternoon measurements showed significantly higher values in MSG and MSGLY compared to the control. Regarding respiratory rate, morning values were similar across treatments (*p* > 0.05), while afternoon measurements showed significant increases in the MSG and MSGLY groups relative to the control (*p* < 0.05). Rump temperature was higher in MSGLY than in the other groups during both periods of the day (*p* < 0.001).

Table 3 presents the results for glucose and total peripheral protein, for which no differences were observed among nutritional groups, nor were there significant interactions (*p* > 0.05).

**Figure 2:**
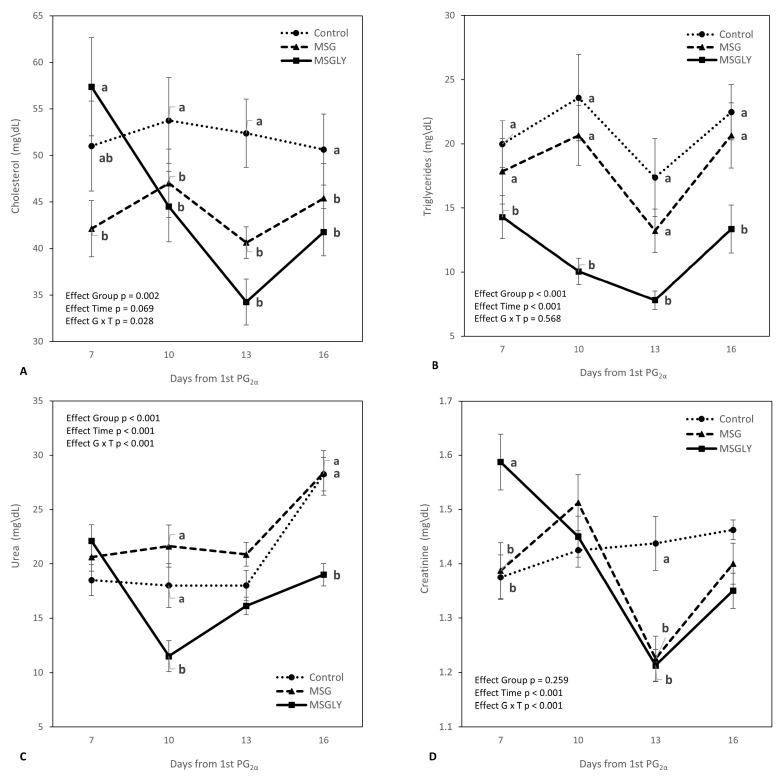
Peripheral cholesterol (**A**), triglycerides (**B**), urea (**C**), and creatinine (**D**), in ewes fed with baseline diet (Control) or supplemented with glutamate monosodium (MSG), or MSG plus glycerin (MSGLY). Data are plotted as mean ± SEM. The *p*-value for the ANOVA effects for group, supplementation interval (effect time), and interaction group vs. time are shown in the figures. ^a,b^ *p* < 0.05.

Figure 2 details the dynamics of cholesterol, triglycerides, urea, and creatinine during the trial. Regarding plasma cholesterol and triglycerides (Figure 2A,B), animals receiving combined supplementation with glutamate and glycerin showed a reduction in the concentrations of these lipid metabolites up to day 13 (time effect, *p* < 0.001). For cholesterol, between days 10 and 16, both MSG and MSGLY groups showed significantly lower levels than the control (G × T interaction, *p* = 0.028). For triglycerides, the MSGLY group showed consistently lower concentrations (*p* < 0.05) throughout the measurement period compared to the other treatments. Significant interactions (*p* < 0.001) between group and time interval were also observed for urea (Figure 2C) and creatinine (Figure 2D). Urea levels increased in the Control and MSG groups between days 13 and 16, whereas in MSGLY, there was a reduction from days 7 to 10, followed by a rise, although always remaining lower than in the other groups. Creatinine showed a different pattern: concentrations decreased in MSGLY from day 7 to day 13, and in MSG from day 10 to 13. In both cases, values were lower than those in the control group (*p* < 0.05).

### 3.3. Ovarian Function Outcomes

#### 3.3.1. Follicular Turnover Before Ovulation Induction

Before the final PGF2α administration (Table 4), there was an increase in the number of large follicles (≥3 mm), total follicle count, and maximum follicular diameter across all groups (time effect, *p* < 0.001). On average, animals in the MSGLY group recorded higher numbers of follicles > 6 mm and greater maximum follicular diameter (*p* < 0.05; Table 4). An interaction between group and time interval was also observed for total follicle number (*p* = 0.034), due to a decrease in the control group after day 11—a phenomenon not seen in the MSG or MSGLY groups.

#### 3.3.2. Follicular Dynamics and Intraovarian Blood Perfusion After Ovulation Induction

In the 48 h following the third and final PGF2α dose (Table 4), all three groups showed a reduction in the number of small follicles (<3 mm) (time effect, *p* = 0.040) and increases in both large follicle count (*p* < 0.001) and total follicle number (*p* = 0.010). The MSGLY group had fewer small follicles (*p* < 0.05) and more large follicles (*p* < 0.05) than the other groups. Both MSG and MSGLY groups exhibited higher total follicle counts compared to the control (*p* < 0.05).

**Figure 3:**
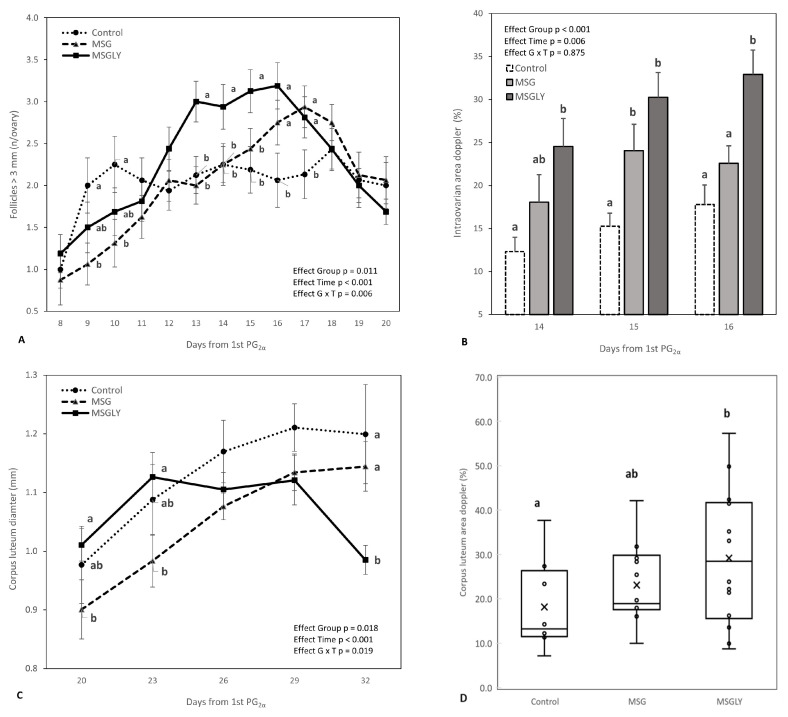
Follicles ≥ 3 mm counted by ultrasonography performed during the experimental interval (**A**); intraovarian perfusion Doppler area measured 48 h after ovulation induction by 3rd PG2α (**B**); diameter of corpus luteum measured on the 20th day to the 32th day after ovulation induction (**C**) and Doppler color area of corpus luteum performed on the 29th day after ovulation induction (**D**), in ewes fed with baseline diet (Control) or supplemented with glutamate monosodium (MSG), or MSG plus glycerin (MSGLY). Data are plotted as mean ± SEM. The *p*-value for the ANOVA effects for group, supplementation interval (effect time), and interaction group vs. time are shown in the figures. ^a,b^ *p* < 0.05.

Figure 3A details the follicular dynamics of follicles > 3 mm throughout the supplementation period. A significant interaction between group and time interval was detected (*p* = 0.006), reflecting the differing trajectories of this follicular class. The MSG and MSGLY groups showed positive, continuous growth during the analysis period, with MSGLY displaying significantly higher values (*p* < 0.05) from day 12 onward. MSGLY peaked on day 16 (48 h post-ovulation induction), while MSG peaked later, on day 17 (72 h post-induction). On the other hand, the control group showed an initial increase between days 8 and 10, followed by a decline on days 11 and 12. Although large follicle numbers rose again in this group on day 18, the values remained consistently lower (*p* > 0.05) than those in the supplemented groups.

Figure 3B shows intraovarian blood perfusion areas measured by Doppler ultrasound during the 48 h following ovulation induction. The MSGLY group exhibited significantly larger perfusion areas (*p* < 0.05) compared to the control throughout the interval and, on day 16, also in comparison to MSG (*p* < 0.05).

#### 3.3.3. Corpus Luteum Growth, Luteal Blood Perfusion Area, and Ovulatory Rate

There was a significant interaction (*p* = 0.019) between group and measurement interval for corpus luteum diameter (Figure 3C), due to differences in growth dynamics. In the MSGLY group, from day 23 onwards, luteal diameter stabilized and then decreased between days 29 and 32, during which it was statistically lower than in the other treatments (*p* < 0.05). In the control and MSG groups, there was continuous progression until day 29. However, the overall mean diameter in the MSG and MSGLY groups was smaller (*p* = 0.018) than in the control group (1.0 ± 0.01 mm vs. 1.1 ± 0.03 mm). The vascularization area of the CL (Figure 3D) in the MSGLY group was greater than in the control (*p* < 0.05) and similar to that in MSG (*p* > 0.05). Ovulation rate (Table 4) was similar (*p* > 0.05) between the supplemented groups and showed a 64% increase (1.8 vs. 1.1) compared to the control (*p* < 0.05).

### 3.4. Expression of Gene Markers

Among the mRNAs encoding genes involved in glutamate uptake and energy metabolism regulation (Table 5), the abundance of three was significantly affected by the group (*p* < 0.05), while no significant differences (*p* > 0.05) were found for SCL1A3, GLUT4, and LEP. The transcript levels of SCL1A1, GRIA1, and GLUD1 were higher (*p* < 0.05) in the MSG group.

Although not statistically significant, the transcript levels of the SCL1A3 gene were very similar between the MSGLY and MSG groups (0.257 vs. 0.248), and both were twice as high (*p* > 0.05) as in the control group (Table 5). This trend was also observed for the LEP gene. For GLUT4, the MSG group showed transcript levels nearly three times higher than those of the other groups (*p* > 0.05).

## 4. Discussion

The evidence collected in the present study confirmed that administration of a fast-release glucogenic precursor such as glycerin enhances the stimulation of ovarian function promoted by monosodium glutamate. The synergistic effect between these two compounds established favorable conditions for improved follicular growth prior to ovulation induction and subsequently supported follicular depletion. This process was accompanied by a substantial increase in the number of growing follicles, greater intraovarian blood perfusion, and, most notably, a significant increase in ovulation rate in the MSGLY group. Studies in small ruminants have shown that both monosodium glutamate [6] and glycerin [14,15,37] favor ovarian activity. Glycerin promotes ovarian function by increasing circulating glucose, which originates from propionic acid produced during ruminal fermentation. In the liver, propionic acid is converted into glucose via hepatic gluconeogenesis from oxaloacetate, or alternatively, glycerin is absorbed through the ruminal epithelium and converted into glucose by the enzyme glycerol kinase [38]. The increase in circulating glucose induces the production of insulin and insulin-like growth factor (IGF-1), both of which act directly on the hypothalamic–pituitary–ovarian axis, enhancing follicular growth and ovulation rate [14]. In the ovaries, follicular growth is supported by the steroidogenic activity of granulosa cells through glucose metabolism, which involves pathways such as phosphatidylinositol 3-kinase, protein kinase B (PI3K–PKB/Akt), and AMP-activated protein kinase (AMPK) [39].

Regarding monosodium glutamate, as the primary excitatory neuromodulatory amino acid (AA) in the central nervous system, glutamate is used as a substrate in various tissues via different metabolic pathways [40]. Positive effects of exogenous glutamate administration on reproductive responses have been reported, largely due to modulation of metabolic and reproductive hormone synthesis [41]. When administered through ruminant feed, extracellular glutamate undergoes minimal catabolism in the rumen and reaches the small intestine, where it is metabolized into AAs such as alanine, aspartate, ornithine, citrulline, arginine, and proline, along with a significant amount of energy that is utilized by enterocytes [1]. Among these, arginine plays a key role in supporting ruminant productivity by promoting growth, reproduction, and lactation [10,42]. In sheep, L-arginine supplementation has been shown to enhance reproductive performance by improving fertility [43], estrus expression, ovulation rate, and prolificacy [44]. The reproductive actions of arginine are mediated by the nitric oxide system, as arginine serves as a precursor for nitric oxide synthesis. Nitric oxide plays an important role in vasodilation and increases blood flow to various organs, including reproductive tissues [45], thereby improving the delivery of nutrients and hormones [45,46]. The nitric oxide system is involved in a variety of reproductive processes, such as regulation of angiogenesis and vascular function, steroidogenesis, hypothalamic–pituitary–gonadal axis signaling, oocyte development, ovulation, and luteolysis across several species [46].

According to Luna-García et al. [4], administration of exogenous glutamate in goats increases serum insulin levels and enhances ovarian activity, resulting in higher ovulation rates. Glutamate is known to interact with ionotropic cell membrane receptors, such as α-amino-3-hydroxy-5-methyl-4-isoxazolepropionic acid (AMPA) and kainate receptors. When activated, these receptors induce membrane depolarization and facilitate calcium ion influx into the intracellular space [40]. Thus, increased extracellular glutamate concentrations can activate AMPA and kainate receptors, stimulating cyclic guanosine monophosphate (cGMP) production, increasing adenosine triphosphate (ATP) levels, and inhibiting ATP-sensitive K^+^ channels. Consequently, the plasma membrane becomes depolarized, leading to insulin secretion by pancreatic β cells [47] Insulin plays a central role in glucose regulation and is also involved in the neuroendocrine regulation of the reproductive axis by stimulating GnRH and LH secretion and enhancing ovarian steroidogenesis [48]. Thus, the results of the present study suggest that the combination of glutamate and glycerin may have stimulated insulin secretion, which, in turn, promotes the release of prostaglandin E2 (PGE2) and increases GnRH secretion [19]. Furthermore, glutamate interacts with astrocytes to enhance PGE2 gliotransmission [49], thereby amplifying GnRH secretion and modulating reproductive processes [16].

The amplification of the ovarian response observed in the MSGLY group was accompanied by significant changes in the peripheral concentrations of lipid metabolites, urea, and creatinine, likely due to increased metabolic effort. This resulted in a physiological response aimed at achieving a new homeostatic balance, reflected in higher temperatures, heart rates, and respiratory rates. The study of the interactions demonstrated how the extra energy released by glycerin led to a greater susceptibility of the animal’s response over the supplementation period and in the presence of higher ambient temperatures (measurements in the afternoon). In any case, the variations recorded never exceeded the critical values for the species, and the animals showed no evident clinical signs of stress throughout the experiment.

Circulating cholesterol levels are correlated with ovarian response in ruminants, as cholesterol is a precursor for steroidogenesis, which is essential for follicular growth [50] and ovulation rate [51]. However, a study in sheep by Kumawat et al. [52] reported that a reduction in cholesterol does not impair ovulation, as compensatory endocrine mechanisms, such as increased circulating insulin, can maintain ovarian activity. In goats, Soares et al. [6] found no differences in cholesterol concentration following administration of 1 g/kg BW of MSG for 20 days. Conversely, Kayode et al. [53] demonstrated that administering 4 mg/kg BW of MSG for 28 days in male rats increased oxidative stress, compromising the cellular redox environment, inhibiting HMG-CoA reductase activity, and significantly decreasing (*p* < 0.05) cholesterol and triglyceride levels. HMG-CoA reductase is a key enzyme in cholesterol synthesis [54], and its inhibition by oxidative stress can affect this process [55]. Furthermore, Kohan et al. [56] observed that MSG can inhibit lymphatic lipid transport, leading to reduced secretion of triglycerides and cholesterol into the lymph in rats. This effect appears to be associated with increased portal transport rather than accumulation in the intestinal lumen, suggesting a complex interaction with lipid metabolism.

Studies have shown that MSG intake can modify the metabolic profile and influence urinary urea and creatinine levels, both of which are key indicators of renal function [57], as MSG has been linked to oxidative stress in renal cells [58]. This oxidative stress may reduce creatine levels and, consequently, circulating creatinine, as observed in small ruminants [59] and consistent with the present findings. Reductions in blood urea levels after glycerin supplementation have also been reported in ruminants [60], an effect likely linked to increased insulin secretion resulting from elevated glucose levels after glycerin fermentation into propionate in the rumen. Insulin promotes protein anabolism and reduces body protein degradation and, consequently, ammonia release. Additionally, improved dietary nitrogen utilization for microbial protein synthesis reduces the amount of nitrogen available for hepatic conversion to urea, thereby lowering blood urea concentrations [60].

Despite the evident reproductive and biochemical outcomes, animals supplemented with glutamate and glycerin did not show increased expression of the amino acid transporters SLC1A1 and SLC1A3 or the glutamate metabolic markers GRIA1 and GLUD1. In fact, transcript levels of these genes were lower in the MSGLY group and similar to the control.

These outcomes have not been previously described in small ruminants or in the expression of the genes evaluated in the present study. In this context, GLUD1 encodes a mitochondrial enzyme involved in the oxidative deamination of glutamate to supply α-ketoglutarate to the tricarboxylic acid cycle [61], while SLC1A1 participates in glutamate and glutamine uptake and is essential for maintaining metabolic homeostasis [62]. Glucose homeostasis depends on the expression levels of the insulin-responsive glucose transporter (GLUT4) in adipocytes, which translocates from intracellular vesicles to the cell surface in response to insulin [63].

In the present study, lower GLUT4 expression was observed in the MSGLY group compared to the control. According to Ripoli et al. [64], diets that lead to increased glucose in mice may reduce the expression of NMDA, a glutamate receptor located in the postsynaptic compartment. In vitro studies have also reported that high circulating glucose levels can downregulate the expression of molecular markers involved in amino acid transport and receptor activity. For example, increased glucose reduced the expression of the L-type amino acid transporter 1 (LAT1) in mouse myoblasts [65], which plays a key role in glutamine export. Therefore, it can be inferred that glycerin supplementation, owing to its strong glucogenic effect, may have compromised glutamate signaling pathways.

However, the expression of certain transporters such as SLC1A3 may be modulated by glutamate concentration, as suggested by Hernández-Melchor et al. [66]. In ruminants, dietary glutamate is primarily metabolized in the small intestine, with minimal absorption into the portal circulation. This indicates that de novo synthesis is required to maintain glutamate homeostasis [1]. Furthermore, amino acids (AA) are crucial regulators of glucose homeostasis, participating in various metabolic processes, such as insulin secretion and gluconeogenesis [67].

Nonetheless, the expression of transporters such as GRIA1 and GLUD1 is not directly influenced by dietary glutamate intake, since glutamate homeostasis is maintained through metabolic pathways [67]. On the other hand, animals supplemented only with MSG did not show the same notable results as the MSGLY group in terms of follicular development, both before ovulation induction and in the 48 h following it. Data on the dynamics of follicles with a diameter > 3 mm indicated that this group experienced continuous quantitative growth throughout the experimental period. However, there was a 24 h delay in the peak frequency of this follicular class, which occurred only 72 h after the last prostaglandin administration. Taken together, these findings support the observed increase in ovulation rate, which was comparable to that of the MSGLY group and 60% higher than the control, confirming an improvement in reproductive efficiency in ruminants through the use of amino acids that act as neurotransmitters, since neuronal activity modulates endocrine and hormonal fluctuations throughout the estrous cycle [5]. Glutamate is the primary neurotransmitter involved in this process, regulated by P4 and E2, and is known to promote neuroendocrine activity during reproduction [68] by stimulating the release of GnRH and, consequently, FSH and LH [69,70]. In goats, supplementation with 1 g/kg BW of monosodium glutamate for 23 days improved intraovarian blood perfusion and increased the number of follicles during ovulation induction [6], while administration of 10 mg/kg BW of MSG in two doses, with a five-day interval, increased follicular number and diameter, intraovarian perfusion, and CL vascularization [3]. In ewes, oral supplementation with 0.5 g/kg BW of MSG for three days prior to mating improved estrus response, duration of estrus, and pregnancy rate after synchronization with progestogen and prostaglandins [7].

As expected, glutamate supplementation led to reduced feed intake and increased rectal temperature and respiratory rate [6,71], and maximized the expression of amino acid transport markers SCL1A1 and glutamate metabolism-related genes GLUD1 and GRIA1. Although the GLUT4 transcript levels were statistically similar, a nearly three-fold increase was observed in the MSG group. It is known that GLUT4 expression can be up- or downregulated depending on physiological states that alter glycemic homeostasis [72]. In this regard, studies have also indicated that glutamate supplementation can improve growth metrics and feed efficiency in ruminants, suggesting that transporter expression may be a potential indicator of nutritional efficacy [10], which could explain the reduced intake observed in the MSG group. However, it has been demonstrated that maternal high-glucose diets can disrupt glutamate homeostasis by increasing extracellular glutamate, which alters transporter expression [73]. This phenomenon may explain why increased expression of transporter genes was not observed in the MSGLY group.

Although both supplemented groups achieved higher ovulation rates, they displayed distinct luteal growth dynamics. In the MSG and control groups, CL diameter continued to increase until day 29. In contrast, in the MSGLY group, CL growth plateaued from day 23 onward, yet blood perfusion was higher in animals receiving both glutamate and glycerin. Glutamate is known to influence luteal quality, mainly through enhanced local blood perfusion [3], which is a key feature of luteal activity, as sufficient P4 production depends on local blood and oxygen supply [74]. A functional CL is defined by at least 30% vascularized area in Doppler ultrasound evaluations and P4 concentrations > 1.0 ng/mL [30]. Thus, although CL size did not increase in the supplemented groups, the higher perfusion observed on day 29 reflects greater luteal activity, which is associated with improved pregnancy rates. Adequate luteal functionality supports endometrial gland differentiation and secretion [75] and promotes endometrial angiogenesis [76], which are essential for embryo implantation and early gestation.

## 5. Conclusions

The inclusion of 150 mL of glycerin in the diet of sheep, beginning eight days prior to ovulation induction, maximizes the stimulation of ovarian function promoted by monosodium glutamate, used as a supplement in sheep feed. These two products act synergistically and effectively, favoring follicular development, intraovarian blood perfusion, ovulation rate, and corpus luteum quality. Based on the results presented, it can be concluded that glycerin and glutamate, when administered at the specified times and dosages indicated in the study, represent an efficient nutritional strategy to optimize ovarian response in sheep.

## Figures and Tables

**Table 1:** Forward and reverse ovine primer sequences, gene bank, and references of genes used in RT-qPCR.

Gene	Length	Direction	Primer (5′ to 3′)	Gene Bank Accession no.	References
*GLUT4*	167	Forward	5′ATCTTTGGCTTCGTGGCCTT	>XM_027974995.3 (*Ovis aries*)	[32]
		Reverse	3′TCCGCCACATACTGGAAACC		
*GRIA1*	121	Forward	5′CTGAACGAGCAGGGGCTTTT	>XM_042250658.2 (*Ovis aries*)	[33]
		Reverse	3′CCACATTGCTGAGGCTGAGA		
*GLUD1*	196	Forward	5′TTGAATGCTGGGGGAGTGAC	>NM_001278567.1 (*Ovis aries*)	[22]
		Reverse	3′CTTGGAACTCTGCTGTGGGT		
*SLC1A1*	183	Forward	5′AGCAACACTGCCTGTCACTT	>XM_004004350.5 (*Ovis aries*)	[34]
		Reverse	3′ATGATCTGCCCAACGCTCAA		
*SLC1A3*	107	Forward	5′TGTTCTCAGAGCCACCACGA	>XM_042233857.2 (*Ovis aries*)	[34]
		Reverse	3′CAGCTCGCATCCCCATCTTT		
*LEPTIN*	189	Forward	5′GTGGACCCCTGTACCGATTC	>XM_027968780.2 (*Ovis aries*)	[35]
		Reverse	3′GCCCAGGGATGAAGTCCAAA		
*RPS18*	174	Forward	5′AGTTCCAGCACATCTTGCGA	>XM_004018745.5 (*Ovis aries*)	[36]
		Reverse	3′GTTCCACCTCGTCCTCAGTG		

**Table 2:** Temperature cycles used in the steps of RT-qPCR reactions.

Stages	Temperature (°C)	Time	
Holding phase	95 °C	10 min.	
Denaturation phase	95 °C	15 seg.	40 cycles
Annealing phase	60 °C	1 min.	
Extension phase	95 °C	15 seg.	
Melting curve phase	60 °C	1 min	
	95 °C	15 seg	

**Table 4:** Follicles turnover and ovarian response in ewes fed with baseline diet (Control) or supplemented with glutamate monosodium (MSG), or MSG plus glycerin (MSGLY).

Parameters	Group				*p*-Value		
	Control	MSG	MSGLY	SEM	Group	Time	G vs. T
Follicle traits before ovulation induction *							
Follicles < 3 mm, *n*/ovary	2.7	2.8	2.6	0.076	0.400	0.184	0.340
Follicles ≥ 3 mm, *n*/ovary	1.9 ^a^	1.5 ^b^	1.9 ^a^	0.068	0.005	<0.001	0.154
Follicle ≥ 6 mm, *n*/ovary	0.1 ^a^	0.1 ^a^	0.2 ^b^	0.018	0.011	0.065	0.229
Total follicles, *n*/ovary	4.6	4.3	4.5	0.068	0.115	<0.001	0.034
Largest follicle size, mm	4.5 ^a,b^	4.2 ^a^	4.7 ^b^	0.086	0.029	<0.001	0.943
Ovarian response after ovulation induction **							
Follicles < 3 mm, *n*/ovary	1.8 ^a,b^	2.0 ^b^	1.5 ^a^	0.090	0.040	0.410	0.783
Follicles ≥ 3 mm, *n*/ovary	2.2 ^a^	2.5 ^a^	3.1 ^b^	0.092	<0.001	0.686	0.770
Follicle ≥ 6 mm, *n*/ovary	0.3	0.4	0.3	0.040	0.699	0.011	0.978
Total follicles, *n*/ovary	4.0 ^a^	4.5 ^b^	4.6 ^b^	0.084	0.010	0.842	0.756
Largest follicle size, mm	5.6	5.7	5.4	0.108	0.590	0.410	0.936
Multiple CL rate, % (*n*/*n*) ***	25 (2/8)	63 (5/8)	63 (5/8)	-	-	-	-
*n*° of CL, *n*/ewe	1.1 ^a^	1.8 ^b^	1.8 ^b^	0.098	0.033	-	-

* Follicle measurements performed on the 7th day to the 13th day of supplementation; ** ovarian response in the 48 h after ovulation induction by 3rd PG_2α_; *** performed 9 days after ovulation induction by 3nd PG_2α_. The *p*-value for the ANOVA effect for group, interval of assessment used (effect time), and interaction group vs. time are shown in the table. ^a,b^ *p* < 0.05 differences between groups.

**Table 5:** Messenger RNA relative abundance of gene markers involved in glutamate and energy regulation in adipose tissue of ewes fed with baseline diet (Control) or supplemented with glutamate monosodium (MSG), or MSG plus glycerin (MSGLY). Arbitrary units defined as the abundance relative to the mean of RPS18 RNA.

Parameters	Group				*p*-Value
	Control	MSG	MSGLY	SEM	Group
*Glutamate markers*					
SCL1A1	0.032 ^a^	0.274 ^b^	0.060 ^a^	0.047	0.029
SCL1A3	0.111	0.248	0.257	0.050	0.457
GRIA1	0.171 ^a^	0.387 ^b^	0.125 ^a^	0.047	0.023
GLUD1	0.093 ^a^	0.409 ^b^	0.194 ^a^	0.057	0.025
*Glucose and energy regulation*					
GLUT4	0.166	0.355	0.108	0.060	0.237
LEP	0.143	0.313	0.242	0.064	0.555

^a,b^ *p* < 0.05 differences between groups.

## Data Availability

The data presented in this study are available on request from the corresponding author due to legal reason.
